# Supplementary material for: Effects of Global Warming on Patients with Dementia, Motor Neuron or Parkinson’s Diseases: A Comparison among Cortical and Subcortical Disorders
Source: Int J Environ Res Public Health. 2022 Oct 18;19(20):13429. doi: 10.3390/ijerph192013429 (PMC9602967; doi:10.3390/ijerph192013429)
Supplement: Supplementary file 1 [file ijerph-19-13429-s001.zip › ijerph-1937767-supplementary.pdf]

Supplementary information - Appendix A: climate change and epidemiological data

Table S1 – Countries climate and epidemiological indices

		$WI_{1990-2016}$	T_{2016}	PD			AD/D			ALS/MND		
				DEATHS	PREV	DALY'S	DEATHS	PREV	DALY'S	DEATHS	PREV	DALY'S
1	Afghanistan	0.052	14.392	0.375	0.270	0.362	0.071	-0.015	0.051	0.318	0.032	0.227
2	Albania	0.055	12.533	0.109	0.140	0.124	-0.034	-0.016	-0.030	-0.072	0.068	-0.150
3	Algeria	0.035	23.650	0.424	0.380	0.424	-0.018	-0.043	-0.024	0.069	0.029	-0.086
4	Andorra	0.034	9.600	0.159	0.121	0.141	-0.032	-0.049	-0.044	0.132	0.210	-0.004
5	Angola	-0.003	22.042	0.502	0.197	0.418	0.237	-0.031	0.154	0.186	0.050	0.093
6	Antigua Barbuda	0.018	26.925	0.145	0.123	0.133	0.006	-0.016	-0.005	-0.057	0.049	-0.047
7	Argentina	0.010	14.450	0.010	0.025	0.018	-0.027	-0.040	-0.036	0.292	0.278	0.220
8	Armenia	0.063	7.142	0.135	0.107	0.131	0.036	-0.005	0.020	0.058	0.037	-0.028
9	Australia	0.009	22.300	0.155	0.082	0.101	-0.060	-0.092	-0.083	0.219	0.303	0.127
10	Austria	0.043	7.592	0.151	0.142	0.146	-0.057	-0.039	-0.058	0.368	0.369	0.172
11	Azerbaijan	0.063	12.917	0.151	0.119	0.138	0.019	0.004	0.010	0.357	0.052	0.242
12	Bahrain	0.063	28.983	0.264	0.290	0.259	-0.011	-0.021	-0.034	-0.076	0.041	-0.105
13	Bangladsesh	0.021	25.725	-0.102	0.250	-0.038	-0.259	-0.006	-0.226	-0.256	0.086	-0.296
14	Barbados	0.017	27.042	0.186	0.113	0.162	0.057	-0.020	0.027	0.127	0.043	0.120
15	Belarus	0.046	7.917	0.108	0.090	0.108	0.013	-0.012	0.004	0.293	0.054	0.177
16	Belgium	0.029	10.767	0.155	0.124	0.136	-0.073	-0.092	-0.088	0.248	0.372	0.109
17	Belize	0.028	26.200	0.304	0.201	0.289	0.046	-0.039	0.027	1.366	0.098	0.891
18	Benin	0.031	28.433	0.156	0.141	0.162	-0.045	-0.071	-0.050	0.229	0.034	0.083
19	Bolivia	-0.006	21.067	0.211	0.141	0.192	0.029	-0.035	0.005	0.241	0.076	0.114
20	Bosnia Herzegovina	0.057	10.583	0.160	0.195	0.180	-0.022	-0.005	-0.021	0.225	0.101	0.166
21	Botswana	0.021	22.800	0.263	0.220	0.253	0.022	-0.032	-0.005	0.425	0.066	0.418
22	Brazil	0.030	25.892	0.150	0.164	0.150	0.001	0.052	0.032	0.773	0.203	0.562
23	Brunei	0.011	27.142	0.179	0.125	0.170	0.027	-0.041	0.008	0.073	0.162	0.073
24	Bulgaria	0.066	11.908	-0.053	0.008	-0.023	-0.014	-0.006	-0.017	-0.285	-0.045	-0.409
25	Burkina Faso	0.023	29.125	0.116	0.144	0.113	-0.036	-0.039	-0.052	0.238	0.051	0.172
26	Burundi	0.010	20.842	0.254	0.159	0.230	0.042	-0.044	0.009	-0.209	0.007	-0.235
27	Cambodia	0.004	28.050	0.576	0.266	0.469	0.178	-0.039	0.114	0.367	0.106	0.206
28	Cameroon	0.016	25.275	0.155	0.116	0.153	-0.002	-0.042	-0.014	0.400	0.015	0.378
29	Canada	0.060	-5.442	0.454	0.430	0.445	-0.014	-0.059	-0.046	0.202	0.421	0.127
30	Cape Verde	0.020	23.358	0.316	0.245	0.303	0.044	-0.038	0.017	0.718	0.082	0.531
31	Central African Rep	0.023	25.550	0.109	0.080	0.105	0.029	0.034	0.042	0.053	-0.006	-0.002
32	Chad	0.032	27.692	0.113	0.120	0.113	-0.042	-0.040	-0.048	0.117	0.052	0.107
33	Chile	0.009	9.075	0.165	0.199	0.175	-0.053	-0.059	-0.072	0.047	0.276	0.002
34	China	0.034	7.442	0.827	1.157	1.040	-0.001	0.056	0.005	-0.011	0.186	-0.121
35	Colombia	0.018	25.483	0.120	0.155	0.134	-0.073	-0.049	-0.068	0.679	0.124	0.417
36	Comoros	0.016	24.617	0.310	0.230	0.291	0.040	-0.041	0.013	-0.038	-0.001	-0.112
37	Costa Rica	0.006	25.158	0.128	0.153	0.140	-0.060	-0.042	-0.058	0.476	0.172	0.486

38	Cote d'Ivore	0.018	27.267	0.208	0.137	0.204	0.031	-0.031	0.022	0.242	0.014	0.204
39	Croatia	0.052	11.992	0.079	0.079	0.086	-0.003	-0.013	-0.009	0.121	-0.040	-0.069
40	Cuba	0.010	26.175	0.079	0.090	0.090	-0.032	-0.048	-0.043	-0.026	0.027	-0.111
41	Cyprus	0.071	20.308	0.049	0.163	0.060	-0.081	-0.017	-0.090	-0.154	0.197	-0.274
42	Czech Republic	0.047	9.192	0.076	0.093	0.075	-0.026	-0.014	-0.033	0.005	-0.008	-0.161
43	Demo Rep Congo	0.010	24.558	0.236	0.067	0.215	0.048	-0.034	0.033	-0.045	-0.011	-0.145
44	Denmark	0.034	9.008	0.567	0.459	0.513	-0.123	-0.201	-0.171	-0.154	0.208	-0.194
45	Djibouti	0.039	28.967	0.382	0.273	0.356	0.064	-0.041	0.033	0.236	-0.001	0.025
46	Dominica	0.020	22.792	0.172	0.155	0.165	0.007	-0.035	-0.018	0.504	0.138	0.519
47	Dominican Rep	0.004	24.567	0.138	0.175	0.146	-0.076	-0.062	-0.080	0.137	0.085	0.003
48	Ecuador	0.003	22.192	0.108	0.136	0.112	-0.063	-0.051	-0.070	0.606	0.076	0.435
49	Egypt	0.057	23.692	0.402	0.407	0.410	-0.015	0.009	-0.008	0.296	0.071	0.074
50	El Salvador	0.019	25.758	0.138	0.186	0.137	-0.063	-0.037	-0.070	0.199	0.077	0.119
51	Eq Guinea	0.017	24.942	0.583	0.423	0.524	0.118	-0.044	0.040	0.003	0.246	-0.149
52	Eritrea	0.049	27.583	0.437	0.269	0.397	0.094	-0.055	0.044	0.172	0.038	0.104
53	Estonia	0.040	6.492	0.056	0.015	0.032	-0.002	-0.016	-0.011	0.001	0.027	-0.125
54	Ethiopia	0.032	23.600	0.407	0.249	0.369	0.093	-0.041	0.060	-0.085	0.039	-0.185
55	Fiji	0.012	24.592	0.054	0.147	0.075	-0.072	-0.007	-0.063	0.335	0.070	0.313
56	Finland	0.053	3.117	0.157	0.058	0.103	-0.023	-0.074	-0.050	-0.238	0.195	-0.242
57	France	0.028	11.792	-0.051	-0.022	-0.049	-0.006	0.011	-0.017	0.050	0.273	-0.061
58	Gabon	0.019	25.833	0.283	0.181	0.258	0.100	-0.020	0.066	0.101	0.002	0.034
59	Georgia	0.064	7.842	0.086	0.052	0.083	0.022	0.002	0.017	-0.064	0.017	-0.063
60	Germany	0.034	9.917	0.145	0.115	0.126	-0.208	-0.177	-0.184	0.013	0.275	-0.099
61	Ghana	0.024	28.225	0.284	0.135	0.265	0.120	-0.030	0.087	0.536	0.062	0.428
62	Greece	0.055	14.842	0.116	0.132	0.115	-0.054	-0.037	-0.054	1.563	0.294	1.149
63	Greenland	0.068	-15.875	0.128	0.127	0.130	-0.020	-0.040	-0.041	0.033	0.298	-0.014
64	Grenada	0.023	27.575	0.293	0.227	0.283	0.051	-0.027	0.028	0.729	0.136	0.645
65	Guatemala	0.026	24.283	0.157	0.180	0.157	-0.026	-0.026	-0.038	0.266	0.057	0.194
66	Guine-Bissau	0.020	27.833	0.162	0.131	0.155	-0.022	-0.044	-0.034	0.182	0.020	0.098
67	Guinea	0.020	26.583	0.179	0.110	0.175	0.015	-0.044	0.002	0.298	0.009	0.118
68	Guyana	0.023	26.475	0.168	0.151	0.160	0.025	-0.016	0.001	-0.015	0.090	-0.085
69	Haiti	0.004	25.533	0.217	0.145	0.207	0.017	-0.063	-0.007	0.166	0.028	-0.017
70	Honduras	0.018	24.475	0.198	0.184	0.187	-0.020	-0.043	-0.036	0.432	0.063	0.255
71	Hungary	0.058	11.483	0.083	0.095	0.090	-0.010	-0.016	-0.014	0.003	-0.002	0.019
72	Iceland	0.048	3.133	0.204	0.134	0.174	-0.001	-0.065	-0.029	0.237	0.251	0.128
73	India	0.025	25.133	0.558	0.297	0.496	0.234	-0.055	0.135	0.569	0.161	0.440
74	Indonesia	0.010	26.417	0.551	0.217	0.434	0.326	-0.001	0.206	0.431	0.106	0.170
75	Iran	0.058	18.558	0.612	0.587	0.625	0.037	-0.012	0.024	0.849	0.073	0.586
76	Iraq	0.054	22.958	0.233	0.227	0.235	-0.016	-0.014	-0.018	0.253	0.036	0.171
77	ireland	0.004	9.700	0.180	-0.041	0.164	-0.050	-0.049	-0.060	0.247	0.376	0.179
78	Israel	0.064	21.192	-0.048	-0.041	-0.056	-0.052	-0.039	-0.061	-0.232	0.174	-0.296
79	Italy	0.044	13.217	-0.055	-0.034	-0.046	-0.018	-0.029	-0.044	0.358	0.345	0.090
80	Jamaica	0.006	25.333	0.208	0.137	0.196	0.015	-0.043	0.001	0.455	0.058	0.317
81	Japan	0.030	11.600	0.102	0.213	0.157	0.102	0.178	0.105	-0.025	0.199	-0.119

82	Jordan	0.065	20.017	0.271	0.287	0.260	-0.038	-0.008	-0.035	0.392	0.045	0.423
83	Kasakhstan	0.051	7.608	0.055	0.090	0.063	-0.042	-0.008	-0.039	-0.022	0.040	-0.060
84	Kenya	0.022	25.575	0.409	0.220	0.364	0.153	-0.021	0.096	0.442	0.031	0.250
85	Kiribati	-0.001	27.492	0.283	0.159	0.256	0.138	-0.012	0.091	0.464	0.047	0.460
86	Kuwait	0.059	26.892	0.428	0.357	0.405	0.001	-0.025	-0.006	-0.210	-0.003	-0.293
87	Kyrgystan	0.055	3.000	0.067	0.052	0.069	-0.002	-0.002	0.002	0.032	-0.001	0.018
88	Laos	0.012	24.217	0.430	0.260	0.428	0.155	-0.015	0.102	0.249	0.111	0.101
89	Latvia	0.035	7.100	0.077	0.076	0.080	0.000	-0.005	-0.004	1.143	0.070	0.852
90	Lebanon	0.067	16.550	0.197	0.339	0.208	-0.125	-0.030	-0.126	-0.277	0.032	-0.324
91	Lesotho	0.026	13.417	0.172	0.180	0.168	0.007	-0.017	-0.009	1.088	0.065	1.062
92	Liberia	0.015	26.175	0.176	0.106	0.171	0.039	-0.035	0.023	0.056	0.004	-0.101
93	Lithuania	0.035	7.767	0.092	0.088	0.096	0.006	-0.008	0.001	1.338	0.213	1.053
94	Luxembourg	0.031	10.108	0.181	0.134	0.160	-0.066	-0.109	-0.097	0.044	0.272	-0.075
95	Macedonia	0.056	11.200	0.059	0.077	0.068	0.012	0.003	0.007	-0.520	0.058	-0.701
96	Madagascar	0.020	22.825	0.172	0.141	0.159	-0.014	-0.031	-0.012	-0.046	0.004	-0.122
97	Malawi	0.013	22.692	0.283	0.190	0.269	0.043	-0.034	0.019	0.152	0.021	-0.098
98	Malaysia	0.013	26.308	0.199	0.264	0.191	-0.076	-0.009	-0.068	0.238	0.086	0.178
99	Maldives	-0.010	27.800	0.258	0.251	0.258	0.012	0.005	0.012	-0.244	0.087	-0.347
100	Mali	0.021	29.142	0.231	0.132	0.200	0.046	-0.037	0.014	-0.057	0.062	-0.130
101	Malta	0.050	20.250	0.153	0.154	0.154	-0.040	-0.045	-0.050	0.145	0.323	0.155
102	Marshall Islands	0.026	28.425	-0.047	0.133	-0.019	-0.159	-0.017	-0.138	0.009	0.047	0.011
103	Mauritania	0.027	28.625	0.205	0.157	0.189	0.030	-0.030	0.004	-0.061	0.033	-0.039
104	Mauritius	0.040	24.017	0.198	0.215	0.181	-0.060	-0.031	-0.065	0.027	0.086	-0.035
105	Mexico	0.023	21.758	0.144	0.177	0.164	-0.062	-0.039	-0.055	0.642	0.098	0.387
106	Moldova	0.064	11.233	0.059	0.065	0.069	-0.035	-0.016	-0.021	0.169	0.011	0.159
107	Mongolia	0.028	0.633	0.052	0.112	0.059	-0.018	-0.002	-0.033	0.384	0.068	0.212
108	Montenegro	0.059	9.642	0.109	0.084	0.103	0.014	0.004	0.013	0.030	0.041	-0.053
109	Morocco	0.046	18.642	0.573	0.573	0.542	0.076	-0.043	0.045	0.425	0.051	0.216
110	Mozambique	0.016	24.600	0.210	0.207	0.207	-0.026	-0.040	-0.041	-0.004	0.071	-0.123
111	Myanmar	0.018	23.683	0.457	0.294	0.412	0.088	-0.031	-0.065	0.267	0.133	0.176
112	Namibia	0.020	21.042	0.257	0.202	0.236	0.022	-0.034	-0.007	0.107	0.048	0.090
113	Nepal	0.039	13.850	0.667	0.295	0.545	0.270	-0.054	0.148	0.458	0.081	0.254
114	Netherlands	0.032	10.583	-0.060	-0.075	-0.670	-0.142	-0.168	-0.159	0.078	0.281	-0.014
115	New Zeland	0.018	10.742	0.185	0.136	0.149	-0.070	-0.084	-0.076	0.108	0.251	0.035
116	Nicaragua	0.010	25.642	0.138	0.159	0.148	-0.050	-0.039	-0.048	0.336	0.042	0.202
117	Niger	0.036	28.333	0.140	0.080	0.131	0.019	-0.028	0.009	-0.050	0.018	-0.195
118	Nigeria	0.026	27.725	0.278	0.198	0.265	0.060	-0.009	0.038	0.043	0.060	-0.018
119	North Korea	0.021	6.592	0.151	0.060	0.144	0.034	-0.038	0.025	0.146	0.026	0.126
120	Northern Mariana Islands	0.021	28.317	0.081	0.140	0.095	-0.043	-0.019	-0.051	0.439	0.055	0.304
121	Norway	0.037	2.233	0.930	0.871	0.939	-0.072	-0.097	-0.093	0.019	0.280	-0.084
122	Oman	0.036	26.383	0.634	0.743	0.679	-0.075	-0.049	-0.087	0.751	0.072	0.433
123	Pakistan	0.047	21.550	0.438	0.279	0.395	0.107	-0.043	0.058	0.664	0.070	0.485
124	Panama	0.009	25.867	0.114	0.143	0.122	-0.082	-0.066	-0.083	0.098	0.094	0.094
125	Papua	-0.003	25.133	0.189	0.157	0.169	0.016	-0.022	-0.007	-0.013	0.069	-0.026

126	Paraguay	0.013	23.325	0.234	0.194	0.231	-0.020	-0.061	-0.033	2.154	0.108	1.776
127	Peru	0.005	20.225	0.159	0.123	0.152	-0.040	-0.075	-0.051	-0.175	0.056	-0.242
128	Philippines	0.010	26.342	0.170	0.169	0.176	0.013	-0.008	0.006	0.170	0.067	0.025
129	Poland	0.045	9.367	0.137	0.142	0.140	-0.066	-0.088	-0.087	0.029	0.065	-0.087
130	Portugal	0.027	15.900	0.343	0.319	0.329	-0.042	-0.043	-0.049	0.957	0.376	0.482
131	Puerto Rico	0.016	25.283	0.141	0.122	0.135	0.013	-0.029	-0.008	0.162	0.080	0.057
132	Qatar	0.062	28.692	0.325	0.340	0.337	-0.042	-0.027	-0.048	-0.039	0.044	-0.100
133	Romania	0.067	10.358	0.081	0.102	0.092	-0.013	-0.009	-0.015	0.244	0.064	0.085
134	Russia	0.060	-4.375	0.090	0.071	0.090	0.000	-0.010	-0.003	0.399	0.027	0.253
135	Rwanda	0.014	19.533	0.525	0.246	0.458	0.162	-0.041	0.110	0.062	0.029	-0.022
136	Saint Lucia	0.020	26.642	0.213	0.180	0.208	0.012	-0.021	0.001	0.615	0.148	0.508
137	Saint Vincent and Grenadines	0.022	27.733	0.171	0.186	0.179	-0.058	-0.046	-0.056	1.762	0.131	1.310
138	Samoa	0.011	27.867	0.085	0.132	0.091	-0.044	-0.017	-0.044	-0.076	0.058	-0.066
139	Sao Tome	0.016	23.458	0.222	0.138	0.195	0.032	-0.035	0.014	0.268	0.028	0.093
140	Saudi Arabia	0.052	25.917	0.657	0.651	0.675	0.038	-0.002	0.025	0.881	0.071	0.482
141	Senegal	0.021	29.042	0.248	0.112	0.222	0.064	-0.060	0.035	0.168	0.021	0.084
142	Serbia	0.063	11.642	0.087	0.076	0.089	0.018	-0.004	0.008	0.225	0.039	0.089
143	Seychelles	0.027	27.892	0.160	0.190	0.151	-0.024	-0.011	-0.030	0.006	0.075	-0.038
144	Sierra Leone	0.018	26.975	0.154	0.127	0.156	-0.003	-0.036	-0.007	0.136	0.028	-0.020
145	Singapore	0.019	28.092	0.113	0.162	0.127	0.032	0.115	0.063	0.099	0.206	-0.012
146	Slovakia	0.054	8.758	0.080	0.097	0.086	-0.006	-0.013	-0.014	0.425	0.061	0.250
147	Slovenia	0.047	9.900	0.067	0.097	0.074	-0.046	-0.024	-0.051	-0.792	-0.201	-0.824
148	Solomon Islands	-0.006	25.575	0.127	0.129	0.132	0.034	-0.005	0.012	0.066	0.054	0.095
149	Somalia	0.015	27.475	0.195	0.138	0.178	-0.004	-0.052	-0.017	-0.056	0.001	-0.161
150	South Africa	0.027	19.033	0.213	0.142	0.199	0.064	-0.031	0.034	0.293	0.059	0.198
151	South Korea	0.024	12.058	0.246	0.210	0.214	-0.026	-0.042	-0.045	-0.339	0.263	-0.347
152	South Sudan	0.033	27.950	0.342	0.159	0.305	0.102	-0.049	0.070	-0.015	0.006	-0.165
153	Spain	0.031	14.283	0.060	0.080	0.049	-0.096	-0.127	-0.124	0.411	0.367	0.121
154	Sri Lanka	0.017	27.758	0.082	0.205	0.119	-0.104	-0.001	-0.074	0.216	0.096	-0.002
155	Sudan	0.049	28.275	0.532	0.382	0.504	0.081	-0.028	0.050	0.340	0.069	0.216
156	Suriname	0.021	26.417	0.201	0.140	0.178	0.073	-0.016	0.036	0.558	0.100	0.395
157	Sweden	0.034	2.925	0.183	0.136	0.151	-0.004	-0.040	-0.027	0.359	0.335	0.253
158	Switzerland	0.036	6.350	0.139	0.103	0.108	-0.017	-0.061	-0.050	-0.074	0.228	-0.129
159	Syria	0.058	19.000	0.418	0.409	0.426	-0.006	-0.025	-0.012	0.234	0.040	0.056
160	Tajikistan	0.056	4.558	0.120	0.089	0.120	-0.022	-0.042	-0.028	-0.019	-0.011	-0.134
161	Tanzania	0.011	23.125	0.352	0.240	0.315	-0.002	-0.059	-0.016	0.114	0.040	-0.034
162	Thailand	0.005	27.350	0.252	0.317	0.276	-0.052	-0.015	-0.052	-0.034	0.100	-0.073
163	The Bahamas	0.016	25.433	0.115	0.094	0.105	0.041	0.005	0.021	-0.052	-0.002	-0.125
164	The Gambia	0.019	28.333	0.182	0.096	0.204	0.044	-0.037	0.026	0.051	0.013	-0.010
165	Timor-Leste	0.001	25.633	0.506	0.266	0.448	0.160	-0.012	0.115	0.397	0.136	0.115
166	Togo	0.032	27.942	0.175	0.151	0.179	-0.017	-0.041	-0.023	0.197	0.011	0.132
167	Tonga	0.008	25.042	0.098	0.139	0.110	0.008	0.000	-0.001	-0.014	0.054	0.001
168	Trinidad Tobago	0.020	26.750	0.108	0.132	0.113	-0.033	-0.029	-0.042	0.065	0.078	0.100
169	Tunisia	0.047	20.867	0.400	0.449	0.430	-0.019	-0.023	-0.023	0.156	0.051	-0.031

170	Turkey	0.067	12.133	0.256	0.338	0.278	-0.072	0.025	-0.041	-0.189	-0.015	-0.361
171	Turkmenistan	0.048	16.808	0.142	0.183	0.162	-0.046	-0.023	-0.043	0.239	0.057	0.118
172	Uganda	0.023	23.475	0.296	0.239	0.286	0.024	-0.042	-0.002	0.173	0.059	0.068
173	UK	0.017	9.192	0.325	0.223	0.263	-0.068	-0.103	-0.084	0.238	0.345	0.002
174	Ukraine	0.064	9.742	0.069	0.057	0.071	-0.013	-0.016	-0.013	0.185	0.018	0.140
175	United Arab Emirates	0.052	28.133	0.486	0.414	0.505	0.019	-0.043	0.002	0.670	0.017	0.498
176	Uruguay	0.007	17.600	0.071	0.109	0.082	-0.052	-0.046	-0.061	-0.015	0.234	-0.042
177	USA	0.042	8.958	0.224	0.095	0.150	0.116	-0.005	-0.038	0.279	0.034	0.143
178	Uzbekistan	0.053	14.308	0.140	0.153	0.155	-0.013	-0.009	-0.013	0.372	0.040	0.259
179	Vanuato	0.004	24.308	0.146	0.144	0.148	0.025	-0.011	0.012	0.143	0.053	0.172
180	Venezuela	0.029	26.458	0.093	0.140	0.110	-0.151	-0.134	-0.154	0.286	0.051	0.275
181	Vietnam	0.011	25.025	0.299	0.252	0.270	0.018	-0.026	-0.004	0.283	0.114	0.140
182	Yemen	0.018	23.742	0.609	0.456	0.581	0.057	-0.045	0.029	0.401	0.051	0.208
183	Zambia	0.004	22.567	0.338	0.204	0.331	0.062	-0.025	0.047	0.542	0.033	0.150
184	Zimbabwe	0.016	22.183	0.559	0.085	0.470	0.401	-0.025	0.312	0.382	-0.014	0.332

Table S1 Data analysed in this work. For the 184 countries that have been analysed, it lists the 1990-2016 warming index (column 3), the 2016 annual average temperature (column 4), and the 1990-2016 variations of patients' deaths, prevalence and DALYs for Parkisons patients (PD; columns 5-6-7), for Alzheimer's Disease and other Dementias (AD/D; columns 8-9-10), and for Amyotrophic Lateral Sclerosis/Motor Neuron Diseases (ALS/MND; column 11-12-13). The temperature values refer to the 2-meter temperature: data have been extracted from the World Bank Climate Change Data Portal (https://climateknowledgeportal.worldbank.org/download-data). The PD indices come from Dorsey et al (2018), the AD/D indices from Nichols et al. (2019), and the ALS/MND from Logroscino et al. (2018).

Table S2 – Clusters' population

	HT-LW	HT-HT	LT-LW	LT-HW
**1**	Antigua Barbuda	Algeria	Angola	Afghanistan
**2**	Bangladsesh	Bahrain	Argentina	Albania
3	Barbados	Belize	Australia	Andorra
**4**	Brunei	Benin	Bolivia	Armenia
**5**	Burkina Faso	Brazil	Botswana	Austria
6	Cambodia	Chad	Burundi	Azerbaijan
**7**	Cameroon	Djibouti	Chile	Belarus
**8**	Cape Verde	Egypt	Dominica	Belgium
9	Central African Rep	Eritrea	Ecuador	Bosnia Herzegovina
**10**	Colombia	Ethiopia	ireland	Bulgaria
**11**	Comoros	Guatemala	Madagascar	Canada
12	Costa Rica	Kuwait	Malawi	China
**13**	Cote d'Ivore	Mauritania	Mexico	Croatia

**14**	Cuba	Mauritius	Namibia	Cyprus
15	Demo Rep Congo	Niger	New Zeland	Czech Republic
**16**	Dominican Rep	Nigeria	North Korea	Denmark
**17**	El Salvador	Oman	Peru	Estonia
18	Eq Guinea	Qatar	Rwanda	Finland
**19**	Fiji	Saudi Arabia	South Korea	France
**20**	Gabon	Seychelles	Tanzania	Georgia
21	Ghana	South Sudan	UK	Germany
**22**	Grenada	Sudan	Uruguay	Greece
**23**	Guine-Bissau	Togo	Zambia	Greenland
24	Guinea	United Arab Emirates	Zimbabwe	Hungary
**25**	Guyana	Venezuela		Iceland
**26**	Haiti			Iran
27	Honduras			Iraq
**28**	India			Israel
**29**	Indonesia			Italy
30	Jamaica			Japan
**31**	Kenya			Jordan
**32**	Kiribati			Kazakhstan
33	Laos			Kyrgystan
**34**	Liberia			Latvia
**35**	Malaysia			Lebanon
36	Maldives			Lesotho
**37**	Mali			Lithuania
**38**	Marshall Islands			Luxembourg
39	Mozambique			Macedonia
**40**	Myanmar			Malta
**41**	Nicaragua			Moldova
42	Northern Mariana Islands			Mongolia
**43**	Panama			Montenegro
**44**	Papua			Morocco
45	Paraguay			Nepal
**46**	Philippines			Netherlands
**47**	Puerto Rico			Norway
48	Saint Lucia			Pakistan
**49**	Saint Vincent and Grenadines			Poland
**50**	Samoa			Portugal
51	Sao Tome			Romania
**52**	Senegal			Russia
**53**	Sierra Leone			Serbia
54	Singapore			Slovakia
**55**	Solomon Islands			Slovenia
**56**	Somalia			South Africa
57	Sri Lanka			Spain

**58**	Suriname	Sweden
**59**	Thailand	Switzerland
60	The Bahamas	Syria
**61**	The Gambia	Tajikistan
**62**	Timor-Leste	Tunisia
63	Tonga	Turkey
**64**	Trinidad Tobago	Turkmenistan
**65**	Uganda	Ukraine
66	Vanuato	USA
**67**	Vietnam	Uzbekistan
**68**	Yemen	

Table S2 list of the names of the countries in the 4 clusters: the high-temperature and high-warming (HT-HW) cluster; the high-temperature and low-warming (HT-LW) cluster; the low-temperature and high-warming (LT-HW) cluster; and the low-temperature and low-warming (LT-LW) cluster.
WBCCDP: World Bank Climate Change Data Portal: link: https://climateknowledgeportal.worldbank.org/download-data.

Table S3

A *AD/D Deaths*	**HT-HW**	**HT-LW**	**LT-HW**	**LT-LW**
**HT-HW**	100.0%	44.8%	20.0%	55.7%
**HT-LW**		100.0%	**1.2%**	98.2%
**LT-HW**			100.0%	7.2%
**LT-LW**				100.0%

B *ALS/MND Deaths*	**HT-HW**	**HT-LW**	**LT-HW**	**LT-LW**
**HT-HW**	100.0%	91.9%	64.4%	50.6%
**HT-LW**		100.0%	44.1%	39.8%
**LT-HW**			100.0%	82.4%
**LT-LW**				100.0%

.***C*** *PD Deaths*	**HT-HW**	**HT-LW**	**LT-HW**	**LT-LW**
**HT-HW**	100.0%	**0.7%**	**0.5%**	9.7%
**HT-LW**		100.0%	29.3%	50.5%
**LT-HW**			100.0%	22.4%
**LT-LW**				100.0%

Table S3. Differences between the distributions of deaths indices of the 4 clusters for AD/D, ALS/MND and PD patients. The table reports the p-value of the Student's t-test calculated between the distributions of deaths for the different clusters. Bold values identify the statistically significant correlations with Student's t-test $p < 0.05$ (5%).

Table S4

A *AD/D DALYs*	**HT-HW**	**HT-LW**	**LT-HW**	**LT-LW**
**HT-HW**	100.0%	57.4%	13.4%	77.6%
**HT-LW**		100.0%	**0.9%**	89.7%
**LT-HW**			100.0%	11.2%
**LT-LW**				100.0%

B *ALS/MND DALYs*	**HT-HW**	**HT-LW**	**LT-HW**	**LT-LW**
**HT-HW**	100.0%	59.5%	67.5%	72.5%
**HT-LW**		100.0%	21.6%	36.5%
**LT-HW**			100.0%	93.1%
**LT-LW**				100.0%

C *PD DALYs*	**HT-HW**	**HT-LW**	**LT-HW**	**LT-LW**
**HT-HW**	100.0%	**0.2%**	**1.0%**	**3.2%**
**HT-LW**		100.0%	30.2%	74.8%
**LT-HW**			100.0%	39.6%
**LT-LW**				100.0%

Table S4. Differences between the distributions of DALYs indices of the 4 clusters for AD/D, ALS/MND and PD patients. The table reports the p-value of the Student's t-test calculated between the distributions of DALYs for the different clusters. Bold values identify the statistically significant correlations with Student's t-test $p < 0.05$ (5%).
